# The Veronesi quadrantectomy: an historical overview

**DOI:** 10.3332/ecancer.2017.743

**Published:** 2017-06-08

**Authors:** Giovanni Corso, Paolo Veronesi, Virgilio Sacchini, Viviana Galimberti, Alberto Luini

**Affiliations:** 1 European Institute of Oncology, Division of Senology, Via G Ripamonti 435, Milan 20141, Italy; 2 Università degli Studi di Milano, Via Festa del Perdono 7, Milan 20100, Italy; 3 European Institute of Oncology, Unit of Molecular Senology, Via G Ripamonti 435, Milan 20141, Italy

**Keywords:** breast cancer, conservative treatment, mastectomy, axillary dissection, radiotherapy

## Abstract

Following a clinical trial in which the Halsted mastectomy was compared to the less invasive quadrantectomy, no differences were reported in terms of local recurrence, disease-free or overall survival between the two. As a result, Umberto Veronesi was the first in the world to state that the radical mastectomy appeared to involve unnecessary mutilation in patients with breast cancer of less than 2 cm and no palpable axillary nodes.

To date, the Veronesi quadrantectomy is routinely considered for breast cancer treatment. This brief review, which highlights the main advances over the last 50 years, is dedicated to Professor Umberto Veronesi.

## Introduction

‘*Some years ago, I purposed, during a scientific meeting, to compare the traditional mutilating mastectomy with a new conservative surgical approach (the so-called quadrantectomy). At this time, I sensed that the radical Halsted mastectomy was not always necessary. This was not well received by the audience and they thought I was a crazy physician*.’

Some years later, following a clinical trial, this evidence was published with great success in the *New England Journal of Medicine*.

No differences were reported in terms of local recurrence, disease-free survival or overall survival (OS) between the Halsted mastectomy and the quadrantectomy surgeries. Veronesi was the first in the world to state that the radical mastectomy appeared to involve unnecessary mutilation in patients with breast cancer of less than 2 cm and no palpable axillary nodes [1].

To date, the Veronesi quadrantectomy is routinely considered for breast cancer treatment. This brief review, which highlights the main advances over the last 50 years, is dedicated to Professor Umberto Veronesi.

## 1950: the beginning

At the end of 1950s, Umberto Veronesi conceived a new approach to the treatment of cancer. At the time, the aim was to give the ‘maximum tolerated treatment’ in the hope of curing disease. However, as treatments and diagnostic techniques were improving, Veronesi realised that a new approach was necessary and developed the new concept of minimum effective treatment’.

The first studies of Umberto Veronesi were focused on the treatment of cutaneous melanoma. The author verified that clinically uninvolved lymph node dissection should not be performed [2], and subsequently, he demonstrated that wide excision (3 cm) is not necessary in the treatment of early melanoma, and in fact, survival was identical in patients receiving a limited resection (1 cm) [3].

## 1969: the new discovery

The modern prospective study of Umberto Veronesi now focused on breast cancer, a much commoner cancer than melanoma and the main cause of cancer death in women worldwide.

The randomised study conceived and conducted by Veronesi was the first to demonstrate that conservative breast surgery plus radiotherapy, which leaves the breast intact, can substitute mutilating mastectomy and yet obtain the same cure rates.

During this period, mammography was being introduced and this was able to detect much small tumours, so that breast cancer could be diagnosed at a much earlier stage. Increasing knowledge about tumour biology and carcinogenesis, opened a new concept of breast cancer treatment, in which the Halsted mastectomy was, in certain cases, an excessive approach (so-called overtreatment).

In 1969, the World Health Organisation in Geneva convened a meeting of investigators to evaluate the methods of diagnosis and treatment of breast cancer. Umberto Veronesi was among the 15 invited experts. He presented a proposal for a randomised trial to compare the traditional mastectomy with a new conservative approach consisting of wide resection (so-called later quadrantectomy), followed by radiotherapy. The proposal stimulated intense discussion which in the end was unexpectly accepted.

## 1973–1980: Milan I trial

The study began in 1971 with a number of clinical tests to verify the feasibility of the treatment, along with its cosmetic and psychological results. Randomisation started in 1973 and the accrual of patients continued until December 1980.

The trial was called *Milan I*: A total of 701 patients with breast cancer were recruited, 349 were randomised to the standard treatment (Halsted mastectomy) and 352 were assigned to the quadrantectomy plus axillary dissection plus post-operative breast radiotherapy (so-called QUART). Eligible patients had infiltrating carcinoma up to 2 cm (cT1N0).

Initially, the term ‘quadrantectomy’ seemed more appropriate for this surgical procedure as the operation maintained the radicality that characterised a mastectomy (removal of skin, subcutaneous tissue, gland, and fascia of the pectoral muscle) [4, 5].

To inform the scientific community about the purpose of the trial an interim report was published in the journal *Cancer* in 1977 [6].

On July 1 1981, the *New England Journal of Medicine* published the first results of the trial. These showed very clearly that there was no difference between the two groups of patients as regards local recurrences and survival [1]. Another important finding of the Milan I study was that radiotherapy had no carcinogenic effect.

The publication in this journal was greeted with great interest by the medical profession worldwide and hailed by the public, especially women, as a more acceptable surgical approach. Several major newspapers and magazines devoted considerable space to the findings; The New York Times, The Washington Post, The Los Angeles Times are just some of the cited newspapers.

## 1985–1987: Milan II trial

However, it was not clear whether the results were due to the surgery or to the radiotherapy. To clarify this point, Veronesi proposed a second randomised trial in 1985 to compare quadrantectomy with tumourectomy combined with complete axillary dissection and radiotherapy [7].

Tumourectomy removed the tumour mass with only a limited margin of surrounding tissue, so that the task of ensuring local control was entrusted mainly to the radiotherapy. This second trial concluded in December 1989 recruited 705 patients from 1985 to 1987. Inclusion criteria were similar to the first study ‘Milan I’ except that the tumour volume could be up to 2.5 cm. instead of 2cm.

The two groups differed significantly in terms of frequency of tumour relapses, although the OS was identical, demonstrating that local failure does not impact on survival.

The conclusion of Milan II trial was that a wide resection such as quadrantectomy was necessary to reduce the risk of local tumour relapse.

## 1987–1989: Milan III trial

The Milan III trial would clarify the impact of radiotherapy within the QUART protocol. It was a randomised study that compared QUART with quadrantectomy plus axillary dissection without radiotherapy (QUAD); 567 patients were enrolled in this trial between 1987 and 1989, 294 were randomised to QUART and 263 to QUAD respectively [8] [9].

The results demonstrated a notable difference in the rate of local relapse. In particular in the QUAD group, a higher frequency of local failure was verified in the pre-menopausal sub-group particularly. Importantly, overall survival did not differ between the two main arms of the study.

In subsequent years, the results of the trial were updated and presented to the scientific press. In addition to the first main trial, two novel randomised studies clarifying the role of both ‘extent of surgical resection’- and of ‘postoperative radiotherapy’, both conducted by Umberto Veronesi, were published [10].

Conclusive data were published in 2002 and the results clearly demonstrated that survival rates in the two groups were absolutely identical [11].

## Conclusions

The Veronesi quadrantectomy represents a great milestone in the treatment of breast cancer, and currently, it is the first conservative protocol scientifically validated.

The rationale of Umberto Veronesi during his long career was the minimum effective treatment obtaining the maximum of results in terms of oncological radicality, offering a high quality of life (QoL) with the best long term survival. Veronesi contributed many other important discoveries in addition to the breast quadrantectomy during his career. Sentinel lymph-node biopsy, intraoperative radiotherapy, mastectomy with skin and nipple areola sparing, neo-adjuvant treatments are just a few examples. To date, several therapeutic approaches are based on this concept, including minimally invasive surgery and more recently robotic surgery.

Alongside these important scientific innovations, the best contribution of Umberto Veronesi in terms of patient care was the attention given to each oncologic patient. He said in one of his interviews: ‘*Don’t rush, stop!.... take care of that patient.*’

Umberto Veronesi died in Milan on 8 November 2016.

We are proud to recognise the Veronesi contribution to the scientific community, and we trust that these results will strengthen our commitment to advancing clinical cancer diagnosis, treatment, research, and QoL for the patient.
